# COVID-19 and Adenovirus Multi-Lobar Pneumonia on CT Scan in a Patient with Repeatedly Normal Chest X-Rays Despite Severe Hypoxia and the Need for Non-Invasive Ventilation

**DOI:** 10.7759/cureus.12955

**Published:** 2021-01-28

**Authors:** Abu Ajela Sreh, Ihab Jameel, Hala Musleh, Vani Shankaran, Salim P Meghjee

**Affiliations:** 1 Gastroenterology and General Medicine, Barnsley Hospital NHS Foundation Trust, Barnsley, GBR; 2 Internal Medicine, University Hospitals of Derby and Burton NHS Foundation Trust, Burton-on-Trent, GBR; 3 Internal Medicine, Barnsley Hospital NHS Foundation Trust, Barnsley, GBR; 4 Diabetes and Endocrinology, Barnsley Hospital NHS Foundation Trust, Barnsley, GBR; 5 Respiratory Medicine, Barnsley Hospital NHS Foundation Trust, Barnsley, GBR

**Keywords:** covid-19, coronavirus, pneumonia, chest x-ray, hypoxia

## Abstract

The British Society of Thoracic Imaging (BSTI) has published clear guidance on the classification of chest X-ray (CXR) findings in coronavirus disease 2019 (COVID-19) patients, which are summarised in four main categories: COVID-classical, COVID-indeterminate, COVID-normal, or non-COVID. We report the case of a 34-year-old lady who is otherwise fit and well. She presented with typical COVID-19 symptoms requiring supplemental oxygen, with normal CXR and COVID-19 reverse transcriptase-polymerase chain reaction (RT-PCR) swab on admission. Her condition deteriorated after 24 hours with severe hypoxia requiring up to 60% oxygen. Repeat CXR was normal, which was followed by computed tomography pulmonary angiogram (CTPA) that ruled out pulmonary embolism; however, CTPA confirmed multi-lobar pneumonia consistent with COVID-19. The patient was admitted to the intensive care unit for non-invasive ventilation (NIV) and ongoing care. Extended respiratory screening confirmed positive COVID-19 antibodies and positive adenovirus swabs. The patient also developed COVID-19 related hepatocellular injury and myocarditis in the absence of other causes. These were treated by a multidisciplinary team, and the patient achieved full recovery after three weeks. This case highlights the fact that normal CXR does not rule out COVID-19 pneumonia even in the severely hypoxic patient requiring NIV. Also, it is important to investigate for other potential causes of hypoxia in a deteriorating patient, such as pulmonary embolism and non-COVID causes of pneumonia.

## Introduction

The novel coronavirus disease 2019 (COVID-19), also known as severe acute respiratory syndrome coronavirus 2 (SARS-CoV-2), is an enveloped, non-segmented positive-sense RNA virus belonging to the beta-Coronaviridae family [1]. COVID-19 has been found to be the cause of severe pneumonia and acute respiratory distress syndrome (ARDS), with a significantly high mortality rate [2]. As of January 2021, the number of confirmed cases of COVID-19 globally is over 85 million and it has affected virtually every territory, other than a few isolated South Atlantic and Pacific island states [3], and the number of deaths from COVID-19 exceeds 1.9 million globally [4]. The definitive test for SARS-CoV-2 is the real-time reverse transcriptase-polymerase chain reaction (RT-PCR) test. It is believed to be highly specific, but with sensitivity reported as low as 60-70% [5] and as high as 95-97% [6]. The primary findings of COVID-19 on chest radiograph and computed tomography (CT) are those of atypical pneumonia [7] or organizing pneumonia [5]. However, imaging has limited sensitivity for COVID-19, as up to 18% demonstrate normal chest radiographs or CT when mild or early in the disease course, but this decreases to 3% in severe disease [8,9].

## Case presentation

A 34-year-old female presented to the Emergency Department on 22/09/2020 with a four-day history of shortness of breath, anosmia, and dry cough. She had no past medical history other than bipolar affective disorder, for which she was taking quetiapine. She reported a family history of asthma, otherwise she never smoked, does not drink alcohol, and has not had any recent travel history or been exposed to anyone who is unwell.

On examination, the patient was alert, talking in short sentences with minimal use of accessory muscles. Initial vital signs on admission showed a respiratory rate of 28 breaths per minute, heart rate of 120 beats per minute, Blood pressure of 140/78 mmHg, a temperature of 37.2 degrees Celsius, and oxygen saturation of 97% on 4 liters of oxygen through a nasal cannula. Her airway was patent, chest examination revealed normal breathing sounds bilaterally, and otherwise there were no stigmata of chronic respiratory disease clinically. Heart sounds were normal with no features of heart failure. Abdominal and gross neurological examinations were unremarkable. Her body mass index was 30.8 kg/m^2^. Calves were soft and non-tender, with no signs of deep venous thrombosis.

**Figure 1:**
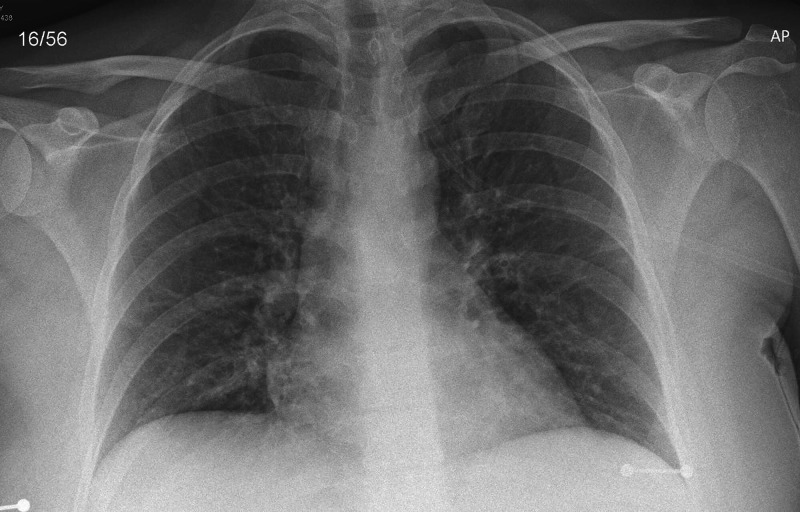
Normal chest X-ray on admission (22/09/2020)

The chest X-ray (CXR) on admission (Figure 1) was normal with no focal abnormalities. As per hospital protocol, she was swabbed for SARS-CoV-2 on admission, which, later on, was negative. She was also tested for Streptococcus pneumoniae PCR and urinary Legionella antigen, and these were also negative. A blood gas analysis on admission revealed compensated type 1 respiratory failure, and she was promptly moved to the respiratory ward for further treatment. Initial electrocardiogram (ECG) showed sinus tachycardia at 120 beats per minute with no features of ischemia or right heart strain.

Initially, the patient was treated as a suspected case of COVID-19 with a potentially associated lower respiratory tract infection. She was started on intravenous antibiotics (clarithromycin), intravenous dexamethasone 6 mg once a day, intravenous fluids for rehydration, and low molecular weight heparin (LMWH) for venous thromboembolism (VTE) prophylaxis.

**Figure 2:**
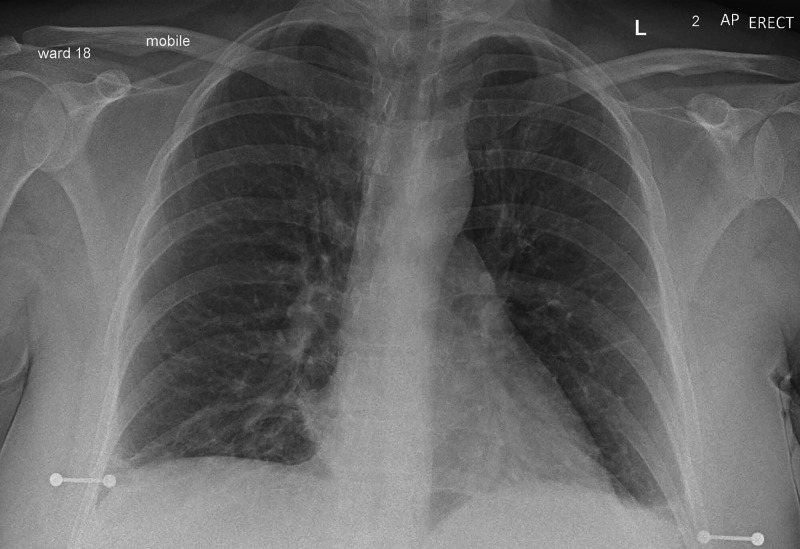
Normal Chest X-ray 26 hours post admission (24/09/2020)

Around 24 hours into her admission, the patient started to deteriorate with respiratory distress and requiring up to 60% of oxygen through a face mask to maintain her peripheral saturation as 98%. Her blood pressure and heart rate were normal at that time. Chest examination revealed mild expiratory polyphonic wheeze and therefore she was given a trial of reversible upper airway treatment with Ipratropium bromide 500 microgram, salbutamol 2.5 mg nebulizers, and intravenous hydrocortisone 100 mg as a stat dose. Repeated portable CXR was performed, which showed no abnormalities (Figure 2).

**Figure 3:**
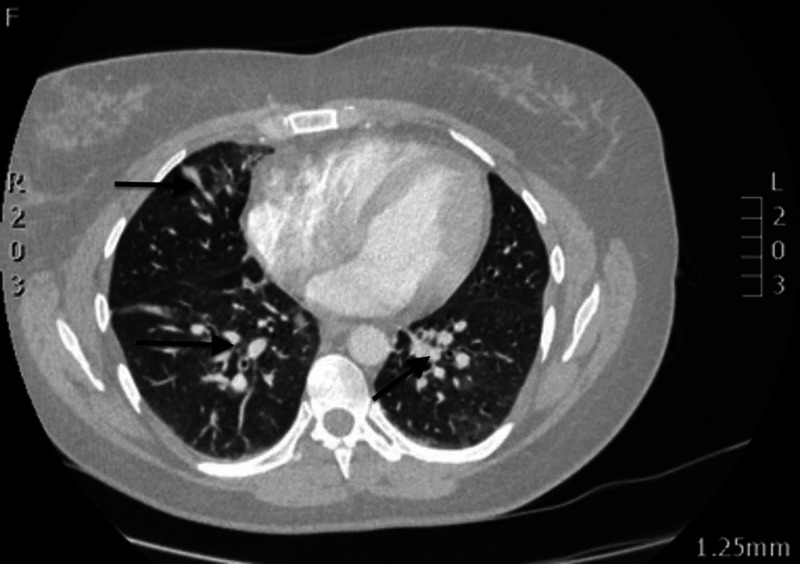
CTPA image (24/09/2020) showing multi-lobar pneumonia changes in the middle zones of both lungs (black arrows) CTPA, computed tomography pulmonary angiogram

**Figure 4:**
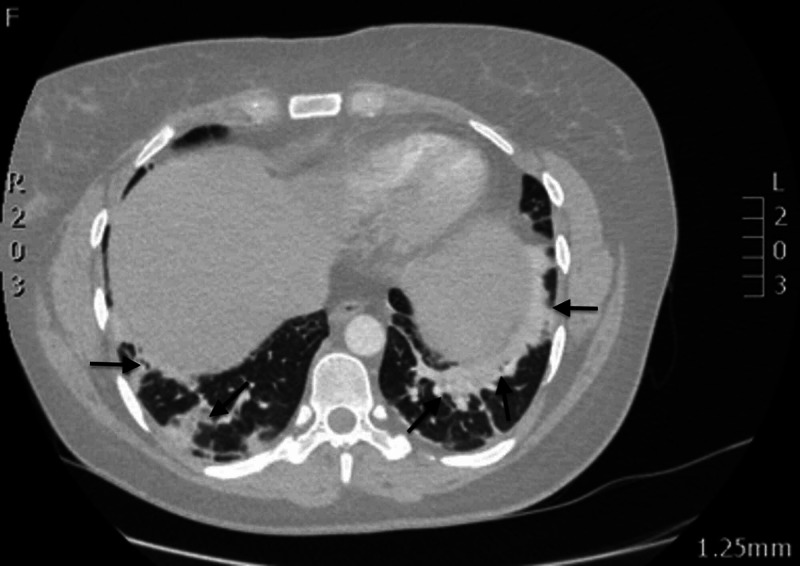
CTPA image (24/09/2020) showing multi-lobar pneumonia in the lower lobes of both lungs (black arrows) CTPA, computed tomography pulmonary angiogram

Giving her degree of hypoxia and clinical deterioration, a CT pulmonary angiogram (CTPA) was performed overnight, revealing multi-lobar pneumonia but excluding a pulmonary embolus (Figures 3, 4). Given the worsening oxygen saturation (92% on FiO_2_ 60%), she was transferred to the intensive care unit (ICU) for non-invasive ventilation in the form of continuous positive airway pressure (CPAP) ventilation at 7 cmH_2_O; alongside continuing intravenous dexamethasone, antibiotics (teicoplanin and clindamycin based on microbiologist advice) were started to cover for secondary bacterial infections.

While in ICU, she tested positive for adenovirus, and a SARS-CoV-2 antibody was positive. The latter test was performed as there was a very strong suspicion of COVID-19 infection despite a negative swab test on admission. The patient developed mild liver enzyme derangement (mild hepatocellular picture) requiring further investigations such as ultrasound liver, viral hepatitis, and autoimmune screen that excluded other liver pathologies. Then, this was concluded as COVID-19 related transaminitis.

She continued to improve, and after five days in ICU, she was deemed well enough to return to the respiratory ward. The patient felt much better clinically and was weaned off oxygen till she was on room air. Also, the patient remained tachycardic at around 110 throughout her admission with mild ankle edema. Echocardiogram showed mild to moderate left ventricular systolic dysfunction with normal valves and no vegetations. Serial cardiac enzymes (troponin) and beta natriuretic peptide (BNP) were normal. The 24-hour ECG showed sinus tachycardia, and normal electrolytes and thyroid function tests. Then repeat echocardiogram after 10 days showed normal left ventricular function. The cardiology team concluded that this was likely COVID-19 associated myocarditis. She received intense physiotherapy sessions and was discharged safely. Follow-ups have been arranged with our respiratory post-COVID clinic, as well as a routine cardiology follow-up with outpatient cardiac MRI.

Investigations

The investigations performed are given in Tables 1-3.

**Table 1:** Full blood count and coagulation screen results APTT, activated partial thromboplastin time

Test	Result	Units	Normal range
Hemoglobin	140	g/L	115–160 (female)
White cell count	15.3	x10^9^/L	4–11
Neutrophils	13.1	x10^9^/L	2–7.5
Lymphocytes	1.3	x10^9^/L	1–3.5
Eosinophils	0.0	x10^9^/L	0.04–0.4
Platelets	245	x10^9^/L	140–415
Prothrombin time	12.1	Seconds	9.1–12.6
APTT	28.2	Seconds	26.0–36.0
D-dimer	<0.5	microgram/L	0.005–0.5

**Table 2:** Renal function, liver enzymes, inflammatory markers, cardiac enzymes, and BNP AST, aspartate transaminase; ALT, alanine transaminase; GGT, gamma-glutamyl transferase; ALP, alkaline phosphatase; PCT, procalcitonin; BNP, beta natriuretic peptide

Test	Result	Units	Normal range
Urea and electrolytes			
Sodium	140	mmol/L	133–146
Potassium	3.5	mmol/L	3.5–5.3
Creatinine	71	umol/L	44–80
Urea	2.7	mmol/L	2.5–7.8
Liver function			
Bilirubin	20	µmol/L	3–22
AST	49	IU/L	14–36
ALT	56	IU/L	9–52
GGT	108	IU/L	0–55
ALP	153	IU/L	38–126
Albumin	42	g/L	32–45
Inflammatory markers			
C-reactive protein	48	mg/L	0–10
PCT	0.18	ng/ml	<0.05
Cardiac enzymes/BNP			
First troponin	<3	ng/L	<3
Second troponin	<3	ng/L	<3
BNP	13	ng/ml	<100

**Table 3:** Arterial blood gas results on admission and after 24 hours

Test	Admission: on 4 liters of oxygen	24 hours: on 40% oxygen	Units	Normal range
pH	7.48	7.42	-	7.35–7.45
PaO_2_	11.8	9.1	kPa	9.5–14.0
paCO_2_	4.1	3.9	kPa	4.5–6.0
HCO_3_	22.3	21.9	mmol/L	22.0–28.0
Lactate	0.8	1.1	mmol/L	<1.0
Glucose	6.2	6.1	mmol/L	4.4–7.8

The extended respiratory micro-organism panel from throat and nose swabs were as follows: SARS-CoV19-2, adenovirus, bocavirus, coronavirus 229E, coronavirus HKU1, coronavirus NL63, coronavirus OC43, human metapneumovirus A+B, influenza A, influenza A H1, influenza A H1N1 pdm09, influenza A H3, influenza B, parainfluenza virus 1-4, respiratory syncytial virus A+B, rhinovirus/enterovirus, *Bordetella pertussis*, *Legionella pneumophila*, and *Mycoplasma pneumonia*. All were negative except for adenovirus positive.

Urinary antigens for legionella and pneumococcal were not detected. HIV antigen and antibody were negative. Urine microscopy and cultures were negative, and blood cultures did not grow any micro-organisms after five days. Routine respiratory sputum cultures were negative. Repeated throat cultures showed no bacteria and *Corynebacterium diphtheriae* not isolated.

## Discussion

The British Society of Thoracic Imaging (BSTI) has published clear guidance on the classification of CXR findings in COVID-19 patients, which are summarized in four main categories: COVID-classical, COVID-indeterminate, COVID-normal, or non-COVID. There are no specific COVID-19 features on CXR; however, classical CXR of COVID-19 refers to ground-glass opacities (68.5%) and multiple, peripheral basal opacities, typically bilaterally (73%) more than unilateral. When the features do not fit into classical or normal, it is classified as indeterminate CXR. When there are other features such as pneumothorax, pleural effusion, or pulmonary edema, it is classified as non-COVID X-rays [8,10,11].

From the beginning of the pandemic, Guan et al. reported back in February 2020 from a cluster of 1,099 patients in China that around 18% of CXR and CT scan can be normal in non-severe cases and around 3% in severe case. The severity score they used is based on the American Thoracic Society (ATS) criteria for classifying the severity of community-acquired pneumonia. Cases were classified as severe if the patient scores one major criterion or three or more minor criteria. Major criteria include septic shock requiring vasopressors or respiratory failure requiring mechanical ventilation. Minor criteria on the other hand include respiratory rate over 30, PaO_2_/FiO_2_ ratio ≤ 250, multilobar infiltrates, confusion, uremia, leukopenia, thrombocytopenia, hypothermia, or hypotension requiring fluid resuscitation. Therefore, there are no clear explanations from this group of patients that any case with a normal CXR was severely hypoxic requiring non-invasive ventilation, similar to what happened with our patient. Also, the timing of the chest imaging is important as it is evident that patients will almost certainly have radiological changes on CT scan following six days of their symptoms. The most common radiological findings on CT scan of COVID-19 are bilateral, subpleural ground-glass appearance, irregular margins. Unfortunately, these initial findings are not organism-specific and can overlap with atypical bacterial or other viral pneumonia such as H1N1 influenza. The pattern of imaging changes will progress as time elapses [8,11].

Also, early reports from London published by Tavare et al. confirmed a small proportion of patients with classical COVID-19 symptoms but negative RT-PCR and normal CXR. However, these patients were well and did not show signs that would require even their admission to the hospital [12].

Another case series from Singapore published by Zhou et al. confirmed that around 40% of patients with COVID-19 PCR positive had initial normal CXR; however, these patients were stable and most of them did not require admission to hospital. One of their patients had initial normal CXR, but as the patient deteriorated and required 35% of oxygen, the repeat CXR started to show features of classic COVID-19 in the form of unilateral consolidation that later on progressed later to bilateral consolidation [13].

Hui et al. studied 358 CXRs performed on 109 COVID-19 patients. Three sub-specialist radiologists assessed these CXRs retrospectively and they concluded that 69 (63%) patients had normal initial CXR on admission. Of these 69 patients, 54 patients continued to have normal CXRs throughout their admission till they were discharged. The remaining 15 patients developed COVID changes on their CXRs after five days. Only four patients, with the initially normal CXR, required supplemental oxygen and two of them required ICU admission and were intubated. Unfortunately, it is not clear whether these two patients developed COVID changes on their repeated CXRs after having normal CXRs on admission [14].

It is very important to rule out pulmonary embolism in severely hypoxic patients; therefore, we proceeded with a CTPA in our patient as she deteriorated overnight. It is well established in the literature that COVID-19 patients are more prone to arterial and venous embolic events. In a study in the Netherlands of 184 patients in intensive care with COVID-19 pneumonia, 31% of their patients developed thromboembolic events [15].

Reports on respiratory co-infections with COVID-19 have been widely published. More evidence supporting worsening disease severity in microbial co-infections is needed. Zhu et al. investigated 257 COVID-19 patients for any other associated 39 respiratory pathogens. They found that 242 (94.2%) patients had co-infection with one or more pathogens (most commonly bacterial infections such as *Streptococcus pneumonia* followed by *Klebsiella pneumonia* and *Haemophilus influenza*). The viral co-infection rate was 31.5%, and the adenovirus co-infection rate in this population was 4.4% (0.4% human bocavirus adenovirus and 4.0% human adenovirus). A total of three patients out of 242 required ICU admission, but, unfortunately, there is no mention of the radiological findings of these patients [16].

## Conclusions

Most patients with COVID-19 do not develop pneumonia, and CXR is normal in up to 63% of patients with mild COVID-19. Seriously sick patients with respiratory symptoms most commonly show CXR features of COVID-19. However; normal CXR does not rule out COVID-19 even in the severe disease requiring ICU admission. Our patient had repeatedly normal CXRs despite her requiring ICU admission for non-invasive ventilation. Also, it is very important to investigate for other potential causes of hypoxia in a deteriorating patient, such as pulmonary embolism, acute pulmonary edema, ARDS, and atypical bacterial causes of pneumonia, as well as non-COVID-19 respiratory viruses such as adenovirus, as in our patient.
